# Associations of All-Cause Mortality with Census-Based Neighbourhood Deprivation and Population Density in Japan: A Multilevel Survival Analysis

**DOI:** 10.1371/journal.pone.0097802

**Published:** 2014-06-06

**Authors:** Tomoki Nakaya, Kaori Honjo, Tomoya Hanibuchi, Ai Ikeda, Hiroyasu Iso, Manami Inoue, Norie Sawada, Shoichiro Tsugane, the Japan Public Health Center-based Prospective Study Group

**Affiliations:** 1 Department of Geography and Institute of Disaster Mitigation for Urban Cultural Heritage, Ritsumeikan University, Kita-ku, Kyoto, Japan; 2 Global Collaboration Center, Osaka University, Suita, Osaka, Japan; 3 School of International Liberal Studies, Chukyo University, Toyota, Aichi, Japan; 4 Epidemiology and Prevention Division, Research Center for Cancer Prevention and Screening, National Cancer Center, Chuo-ku, Tokyo, Japan; 5 Public Health, Graduate School of Medicine, Osaka University, Suita, Osaka, Japan; 6 Graduate School of Medicine, The University of Tokyo, Bunkyo-ku, Tokyo, Japan; University of Westminster, United Kingdom

## Abstract

**Background:**

Despite evidence that neighbourhood conditions affect residents' health, no prospective studies of the association between neighbourhood socio-demographic factors and all-cause mortality have been conducted in non-Western societies. Thus, we examined the effects of areal deprivation and population density on all-cause mortality in Japan.

**Methods:**

We employed census and survival data from the Japan Public Health Center-based Prospective Study, Cohort I (n = 37,455), consisting of middle-aged residents (40 to 59 years at the baseline in 1990) living in four public health centre districts. Data spanned between 1990 and 2010. A multilevel parametric proportional-hazard regression model was applied to estimate the hazard ratios (HRs) of all-cause mortality by two census-based areal variables —areal deprivation index and population density—as well as individualistic variables such as socioeconomic status and various risk factors.

**Results:**

We found that areal deprivation and population density had moderate associations with all-cause mortality at the neighbourhood level based on the survival data with 21 years of follow-ups. Even when controlling for individualistic socio-economic status and behavioural factors, the HRs of the two areal factors (using quartile categorical variables) significantly predicted mortality. Further, this analysis indicated an interaction effect of the two factors: areal deprivation prominently affects the health of residents in neighbourhoods with high population density.

**Conclusions:**

We confirmed that neighbourhood socio-demographic factors are significant predictors of all-cause death in Japanese non-metropolitan settings. Although further study is needed to clarify the cause-effect relationship of this association, the present findings suggest that health promotion policies should consider health disparities between neighbourhoods and possibly direct interventions towards reducing mortality in densely populated and highly deprived neighbourhoods.

## Introduction

In the field of study on social disparities in health, interest is growing in the relationships between neighbourhood conditions and health [1], [2], likely owing to initial findings of associations between mortality and various census-based deprivation indices that reflect the socio-economically disadvantaged conditions of small areas [3], [4]. Variations in health as a function of areal deprivation and socio-economic status have been widely reported in both cross-sectional and prospective studies conducted in many Western societies [5]–[7]. In particular, multilevel modelling has been used to determine whether areal factors affect health outcomes independent of individual socio-economic factors. Despite large variations in study design, numerous multilevel studies have identified significant areal effects [8]–[10]. Meijer et al. [10] conducted a meta-analysis using 18 multilevel studies to examine the areal contextual effects on mortality. They identified that such areal effects are generally salient for smaller units, particularly those with less than 5,000 residents per neighbourhood area in high-income Western societies. Some argue the cause of such effects derives from mediating factors between neighbourhood conditions and individual health [1], [2]. Specifically, the undesirable physical and social environments of deprived neighbourhoods (e.g. insufficient material resource allocation, weak social relationships, and poor reputation) are believed to promote unhealthy behaviours and substantial stress, leading to elevated morbidity and mortality among residents [1], [2].

The health disparities between urban and rural areas have also been considered an important aspect of contextual differences in health [11], [12]. Urbanicity/rurality is often measured using a simple census variable, such as population density [11], [12], or through administratively defined regional divisions [13], [14]. Chaix et al. [11] noted the lack of multilevel studies that compared urbanicity (population density) and deprivation simultaneously by controlling for individual socio-economic status. Thus, they [11] examined whether areal socio-economic status and population density affected cardiovascular mortality in a prospective study of a Swedish parish; results showed that after controlling for individual factors, population density had the dominant effect on mortality. A similar prospective study on all-cause mortality was replicated for all of Denmark [12]: findings suggested that population density was a more consistent contextual indicator than was areal socio-economic variables after controlling for individual factors through multilevel survival analysis. In both cases, higher population density was associated with higher mortality, supporting the ‘urban penalty’ hypothesis that life characteristics in urbanised areas, such as higher exposure to environmental pollution, individualistic lifestyles, and unhealthy behaviours (e.g. poor diet due to better access to fast-food retails) may lead to a higher mortality risk in urban inhabitants. It is also known that associations between area deprivation and population health tend to be stronger in urbanised areas in Canada [14] and the UK [15], [16]. This may suggest that ‘urban penalty’ is most intense in disadvantaged areas within highly urbanised regions.

In contrast to Western societies, there is little information regarding the neighbourhood-level areal contextual effects on mortality in non-Western societies, such as Japan, which has the longest life expectancy in the world. Researchers in Japan have noted significant relationships between mortality rate and various areal socio-economic indicators (e.g. average income and unemployment) [17]–[20]. However, these have been demonstrated only at the prefectural or municipal level (i.e. in broader geographical units than the neighbourhood) and all of these studies were cross-sectional and ecological (i.e. associations between variables aggregated by areal units). Mortality was not directly examined in these recent neighbourhood-level epidemiological studies in Japan; rather, areal aggregates of survey data were used to uncover possible associations between various health outcomes (self-rated health [21], [22], mental health [23], [24], and physical activities [25]) and neighbourhood contextual factors such as social cohesion, perceived walkability, and food access. Additionally, one prospective study [26] examined the effects of areal agglomeration of subjective stress on mortality at the municipal level, also using aggregate data. While aggregating survey information can be useful for constructing areal contextual variables, census-based indicators are superior because of their wider applicability to different regions at various scales [11]. Therefore, the use of census-based variables to estimate areal contextual effects at the neighbourhood level would yield better geographical descriptions of areal health disparities; furthermore, such descriptions would be useful in the planning of health promotion interventions to diminish social and areal health disparities [27].

Thus, in the present study, we examined the simultaneous effects of neighbourhood deprivation and population density on all-cause mortality in a Japanese population by using multilevel models that controlled for individual socio-economic factors, just as the Swedish and Danish studies did [11], [12]. Using the cohort dataset of middle-aged Japanese population in non-metropolitan areas, our primary aim was to examine whether living in more deprived and crowded neighbourhoods increased mortality risk. We also examined possible interactions of the two areal factors. Consistent with the aforementioned observations in the UK and Canada, it was expected that the impact of area on health would be greater in urbanised than in rural regions.

## Materials and Methods

### 1. Survival Data

We employed survival data taken from Cohort I of the Japan Public Health Center-based Prospective (JPHC) Study. The JPHC Study began in January 1990 with a baseline survey (self-administered questionnaire) that was distributed to registered subjects aged 40 to 59 years who lived in one of four public health centre (PHC) districts: Ninohe PHC in Iwate prefecture, Yokote PHC in Akita prefecture, Saku PHC in Nagano prefecture, and Ishikawa PHC in Okinawa prefecture. The number of respondents was 43,149 (20,665 men and 22,484 women; response rate: total 79%, men 76%, women 82%). Of them, 9 were excluded because they were deemed ineligible (7 because they were of non-Japanese nationality and 2 because they moved before the start of the study). We also excluded people with a baseline history of cancer, stroke, or cardiovascular disease (*n* = 1,549) because the presence of such high-risk diseases may have influenced individual socio-economic status and living area. This left 41,591 remaining subjects. The PHC districts were less urbanised, middle-sized cities outside metropolitan areas. More details of the JPHC Study design are described elsewhere [28], [29]. The study was approved by the human ethics review committee of the National Cancer Center, Japan.

Eligible subjects' residential addresses at baseline were geocoded to identify their living areas in *chocho-aza* (CA) units, which were used for the 1995 population census of Japan. This is the smallest administrative unit, roughly comparable to a European parish or a US block group. We excluded four subjects whose addresses could not be geocoded, and a further 263 because detailed census information was not provided by the statistical bureau for their CAs or their CAs had a small number of households (<25), which we avoided using because they were considered statistically unstable areal indicators. The Statistical Bureau of Japan occasionally masks detailed tabulated numbers in censuses to protect the confidentiality of sensitive residential information; another subject was excluded from our dataset for this reason. The remaining 41,324 were potentially eligible subjects with appropriate census-based indicators. Of them, we excluded 3,869 who did not provide information on the individual attributes used for this analysis at the baseline survey. The final dataset consisted of 37,455 subjects (18,008 men and 19,447 women) living in 263 CAs. The endpoints for each subject were all-cause mortality. The study period was from January 1, 1990 to December 31, 2010. Person-years for each subject were calculated according to follow-up data from the baseline until the subjects' date of death or the end of the follow-up period, whichever came first.

### 2. Neighbourhood-level factors

We constructed areal indicators for each of the 263 CAs by using the 1995 census, which firstly introduced the small area tables and geographic information system (GIS) boundary file for each CA. The medians of area size, population, and household numbers in the studied CAs were 1.17 km^2^, 415, and 122, respectively. Areal indicators were used to evaluate the effects of neighbourhood urbanicity and deprivation on mortality. We used population density as the areal indicator of urbanicity. To compute it, we used the CA area sizes obtained from the census GIS boundary information and the total CA population size obtained from the census tables.

Regarding deprivation, we used a composite indicator of various census-based variables, a common choice for summarizing deprivation in small areas [30]. We employed the areal DI (ADI) derived by one of the authors [31], consisting of weighted sums of a number of census-based variables calculated using the same method as the Breadline Britain poverty measure [32], [33] and the European transnational ecological deprivation measure [34]. See [Appendix S1] for details.

The areal indicators for this analysis were converted into variables consisting of quartile categories ranging from 1 (*lowest density/least deprived*) to 4 (*highest density/most deprived*) for population density and ADI, respectively.

### 3. Individual-level factors

The JPHC baseline survey provides data on a large variety of individual-level attributes. We used the continuous and dichotomous variables of age and sex, respectively, as basic demographic covariates. Proxy variables for socio-economic status were education and occupation at baseline. We defined three categories for education: junior high school, senior high school, and higher education (‘college or vocational school’ and ‘university’). Regarding occupation, each subject was asked to describe his/her current occupation in an open-answer format; these data were then grouped into ten categories following the thrid revision of Japan Standard Occupational Classification (JSOC) used by the 1995 population census of Japan: professionals, administrators, office clerks, sales clerks, service workers, security workers, agricultural workers, transportation & communication workers, manual labourers, and not working. Appendix S2 shows the correspondence between the JSOC-based classification used in this study and the major groups of the International Standard Classification of Occupations (ISCO-88) proposed by the International Labour Organisation. While men who indicate ‘not-working’ are typically unemployed, women who select this option typically work in the home (i.e. housewives). Thus, considering the similarities in census categories and the differences in ‘not-working’ by gender, we created six original categories: white collar (professionals, administrators, and office clerks), grey collar (sales clerks, service industry workers, and security guards), agricultural workers, blue collar (transportation or communication industry workers, and manual labourers), not-working men, and not-working women.

From the JPHC baseline, we included various other categorical variables [behavioural habits (drinking, smoking, and regular exercise) and body mass index (BMI)] and two dichotomous variables on previously diagnosed diabetes and hypertension, as areal variations in these factors are possible confounders or mediating factors in the association between areal characteristics and mortality. Table 1 summarizes the sample characteristics for individual attributes.

**Table 1:** Characteristics of study subjects.

			Deprivation		Population density	
Factor and category	n	%	Less (Q1 & Q2)	More (Q3 & Q4)	Lower (Q1 & Q2)	Higher (Q3 & Q4)
**Sex**						
men	18,008	48.1%	47.9%	48.3%	48.2%	47.9%
women	19,447	51.9%	52.1%	51.7%	51.8%	52.1%
**Age**						
40s	18,771	50.1%	49.8%	50.4%	49.0%	51.2%
50s	18,684	49.9%	50.2%	49.6%	51.0%	48.8%
**Cohabiting**						
with others including spouse	30,463	81.3%	80.7%	75.7%	77.4%	79.6%
with others but not including spouse	7,397	19.7%	16.8%	21.3%	20.5%	17.0%
living alone	1,078	2.9%	2.6%	3.0%	2.0%	3.4%
**Education**						
junior high school	19,080	50.9%	45.8%	56.2%	57.0%	44.8%
high school	13,902	37.1%	41.8%	32.3%	34.8%	39.5%
higher education	4,473	11.9%	12.4%	11.5%	8.2%	15.7%
**Occupation**						
white collar	6,697	17.9%	19.4%	16.3%	13.6%	22.2%
grey collar	6,350	17.0%	17.5%	16.4%	12.6%	21.3%
agriculture	7,719	20.6%	19.1%	22.2%	34.1%	7.0%
blue collar	10,792	28.8%	29.7%	27.9%	29.7%	27.9%
not working (men)	552	1.5%	1.2%	1.8%	0.9%	2.0%
not working (women)	5,345	14.3%	13.1%	15.4%	9.1%	19.5%
**Body mass index**						
25.0–30.0	9,767	26.1%	22.9%	29.4%	24.9%	27.2%
>30.0	999	2.7%	1.8%	3.5%	2.0%	3.4%
**Smoking habit**						
current	10,618	28.3%	29.4%	27.3%	28.6%	28.2%
**Alchohol intake**						
none	18,714	50.0%	46.0%	54.1%	49.6%	50.3%
>450 (ethanol g per day)	2,988	8.0%	9.3%	6.6%	8.5%	7.5%
**Regular sports habit**						
almost none	27,005	72.1%	71.3%	72.9%	73.4%	70.8%
almost everyday	1,478	3.9%	3.8%	4.1%	3.4%	4.5%
**Previously diagnosed diseases**						
diabetes	1,398	3.7%	3.8%	3.7%	3.9%	3.5%
hypertension	5,310	14.2%	15.2%	13.1%	14.7%	13.6%
**Total**	37,455					

Qs  =  Quartiles, data source: Japan Public Health Center-based Prospective Study, Cohort I.

### 4. Statistical analysis

We estimated hazard ratios (HRs) and 95% confidence intervals (CIs) for all-cause mortality by applying a multilevel parametric proportional hazard regression model with a Weibull distribution and a gamma shared frailty function to the JPHC survival dataset. The shared frailty survival model corresponds to a mixed regression model with a random intercept term for survival data. Model fitting procedures were carried out using the streg command of STATA 13 [35]. Since the estimation of the shared frailty model was unstable for sex-stratified models due to the relatively small number of deaths, we report only the modelling result using sex-combined data. For all of the fitted models in this study, we included the dichotomous variable of sex. The interaction terms of sex with other variables (e.g. in the case of ADI, terms of ADI for men and women are separately created) are also included at both the individual and areal levels in cases where the terms were significant at the 5% level. AIC (Akaike Information Criterion) is also reported for model comparison of fitted survival models.

We assumed two areal levels: the PHC district level (covering broad regional differences in living situation, adjusted for by including dummy variables of PHC district for each subject) and the CA level (covering possible clustering tendencies within the same neighbourhood according to the random intercept of the shared frailty function). The district dummy variables and the random effect were included for all of the fitted models. When we fitted a model, we first controlled for basic individual factors and adjusted for age and sex (Model 0). We then added education, occupation, and co-habitation of subjects (Model 1). In this study, the variables of education and occupation were regarded as individual socio-economic status indicators. The same occupational categories are used in the ADI. Furthermore, since some types of households (e.g. retired head living alone and single parent) are likely to be associated with poverty [31], we adjusted for the co-habitation variable as an aspect of individual deprivation in Model 1. The factors associated with co-habitation were also considered when the ADI was constructed. Second, we added the two census-based areal indicators of neighbourhood, ADI and population density, to Model 1 (producing Model 2) in order to assess the additional contributions of these areal factors to mortality. We further adjusted previously diagnosed hypertension and diabetes, and lean (BMI < 18.5) and obese (BMI > 30) status at baseline as possible confounders of initial health condition (Model 3).

In addition, based on Model 3, we performed the following two analyses: (1) We examined whether alcohol drinking, smoking, and sports activity mediate the association between areal factors and mortality by adding these behavioural variables as covariates (Model 4); (2) we investigated the interactions of the two areal factors by using the interaction term of every quartile category for ADI and population density (Model 5).

## Results

During the 21-year study period, there were 4,666 (men: 3,112, women: 1,554) deaths and 130 (men: 68, women: 62) loss-of-follow-ups among the 37,455 subjects. The results of the fitted survival models are summarised in Tables 2–5. Table 2 shows the estimated HRs and other model statistics from Models 0–2. The base model (Model 0) includes only age and sex, along with the district dummy variables and their thetas; the estimated variance indicates that there was a small extra variation in mortality between neighbourhoods in the study regions. Inclusion of the socio-economic factors (Model 1) resulted in better performance (much smaller AIC compared to Model 0). Although the size of the extra area-level variation is small, Model 1 did not substantially change the size compared to Model 0, indicating that the compositional effects of occupation, education, and co-habitation are not strongly associated with neighbourhood-level variation in mortality in this study. By contrast, including the two areal-level variables (Model 2) reduced a substantial part of the extra area-level variation. These results indicate a significant gradient in mortality along the degrees of both ADI and population density. The most deprived and most densely inhabited neighbourhoods have about 1.144 and 1.214 times higher HRs for all-cause mortality compared with the least deprived and least densely inhabited ones, respectively. Furthermore, the ADI and population density were positively but weakly correlated at this level (cross-product correlation coefficient weighted by sample size: 0.232, p<0.01). Thus, these two factors exerted relatively independent effects on geographical variation in mortality.

**Table 2:** Hazard ratios and model statistics of the shared frailty survival models (Models 0–2).

	Model 0	Model 1		Model 2		
Factor and category	Estimate	Estimate	(95% CI)	Estimate	(95% CI)	trend p
**Deprivation**						0.0322
Q1 (least)				1.000	Reference	
Q2				1.028	(0.935, 1.130)	
Q3				1.147	(1.031, 1.277)	
Q4 (most)				1.144	(0.987, 1.326)	
**Population density**						0.0009
Q1 (lowest)				1.000	Reference	
Q2				1.113	(1.017, 1.218)	
Q3				1.135	(1.030, 1.250)	
Q4 (highest)				1.214	(1.087, 1.355)	
**Education**						
higher education		1.000	Reference	1.000	Reference	
high school		1.024	(0.915, 1.146)	1.031	(0.921, 1.154)	
junior high school		1.149	(1.024, 1.288)	1.161	(1.035, 1.302)	
**Occupation**						
white collar		1.000	Reference	1.000	Reference	
grey collar		1.129	(1.007, 1.265)	1.126	(1.006, 1.262)	
agriculture		1.131	(1.015, 1.260)	1.148	(1.029, 1.282)	
blue collar		1.170	(1.057, 1.294)	1.172	(1.059, 1.296)	
not working (men)		2.254	(1.920, 2.646)	2.247	(1.914, 2.637)	
not working (women)		1.709	(1.498, 1.950)	1.702	(1.492, 1.943)	
**Cohabitating**						
with others including spouse		1.000	Reference	1.000	Reference	
with others but not including spouse		1.314	(1.222, 1.414)	1.314	(1.221, 1.413)	
living alone		1.343	(1.148, 1.572)	1.342	(1.147, 1.570)	
**Model statistics**						
Theta	0.0050	0.0046		0.0000		
Explained geographical variance	0.0%	7.8%		100.0%		
p value for Theta = 0	0.127	0.153		0.499		
Log likelihood	−15,536.8	−15,408.8		−15,401.0		
AIC	31,089.7	30,853.6		30,849.9		
Difference of AIC	0.0	236.1		239.7		

Estimate: estimated hazard ratios for the factors and estimated values for the model statistics (Model 0–2: adjusted by age, sex, and public health centre district); Qs  =  Quartiles, CI  =  confidence interval; trend p: p value of trend test; Explained geographical variance: percentage of reduction in Theta of fitted models compared to Model 0; Difference in AIC: the subtraction of AIC of fitted model from AIC of Model 0.

**Table 3:** Hazard ratios and model statistics of the shared frailty survival models (Models 3 and 4).

	Model 3			Model 4		
Factor and category	Estimate	(95% CI)	trend p	Estimate	(95% CI)	trend p
**Deprivation**			0.0201			0.0172
Q1 (least)	1.000	Reference		1.000	Reference	
Q2	1.022	(0.929, 1.123)		1.018	(0.925, 1.120)	
Q3	1.142	(1.026, 1.271)		1.136	(1.020, 1.264)	
Q4 (most)	1.160	(1.001, 1.344)		1.166	(1.007, 1.352)	
**Population density**			0.0017			0.0054
Q1 (lowest)	1.000	Reference		1.000	Reference	
Q2	1.116	(1.020, 1.221)		1.110	(1.015, 1.215)	
Q3	1.129	(1.024, 1.244)		1.109	(1.007, 1.222)	
Q4 (highest)	1.205	(1.079, 1.345)		1.183	(1.060, 1.321)	
**Education**						
higher education	1.000	Reference		1.000	Reference	
high school	1.021	(0.913, 1.143)		0.994	(0.888, 1.113)	
junior high school	1.149	(1.024, 1.289)		1.092	(0.973, 1.224)	
**Occupation**						
white collar	1.000	Reference		1.000	Reference	
grey collar	1.143	(1.020, 1.280)		1.103	(0.984, 1.236)	
agriculture	1.191	(1.066, 1.329)		1.166	(1.044, 1.301)	
blue collar	1.209	(1.093, 1.338)		1.158	(1.047, 1.282)	
not working (men)	2.289	(1.950, 2.687)		2.171	(1.848, 2.552)	
not working (women)	1.690	(1.481, 1.929)		1.608	(1.409, 1.836)	
**Cohabitating**						
with others including spouse	1.000	Reference		1.000	Reference	
with others but not including spouse	1.316	(1.223, 1.416)		1.283	(1.193, 1.380)	
living alone	1.338	(1.144, 1.565)		1.252	(1.070, 1.465)	
**Model statistics**						
Theta	0.0000			0.0000		
Explained geographical variance	100.0%			100.0%		
p value for Theta = 0	0.499			1.000		
Log likelihood	−15,267.8			−15,087.2		
AIC	30,593.6			30,254.4		
Difference of AIC	496.1			835.2		

Estimate: estimated hazard ratios for the factors and estimated values for the model statistics (Models 3: adjusted by age, sex, public health centre district, histories of diabetes and hypertension, and body mass index; Model 4: adjusted by age, sex, public health centre district, histories of diabetes and hypertension, body mass index, smoking, alcohol intake, and regular sports habit); Qs  =  Quartiles, CI  =  confidence interval; trend p: p value of trend test; Explained geographical variance: percentage of reduction in Theta of fitted models compared to Model 0 shown in Table 2; Difference in AIC: the subtraction of AIC of fitted model from AIC of Model 0 shown in Table 2.

**Table 4:** Hazard ratios and model statistics of the shared frailty survival model with the interaction terms of the two areal factors (Model 5A).

Factor and category		Estimate	(95% CI)	trend p
**Population density × deprivation**				
Population density	Deprivation			
Q1 (lowest)				0.5263
	Q1 (least)	1.128	(0.880, 1.445)	
	Q2	1.000	Reference	
	Q3	1.007	(0.879, 1.154)	
	Q4 (most)	1.026	(0.839, 1.255)	
Q2				0.6922
	Q1 (least)	1.033	(0.883, 1.208)	
	Q2	1.045	(0.895, 1.221)	
	Q3	1.210	(1.029, 1.423)	
	Q4 (most)	1.067	(0.712, 1.599)	
Q3				0.0714
	Q1 (least)	1.106	(0.928, 1.318)	
	Q2	1.030	(0.885, 1.199)	
	Q3	1.323	(1.082, 1.617)	
	Q4 (most)	1.272	(1.028, 1.574)	
Q4 (highest)				0.0004
	Q1 (least)	1.023	(0.856, 1.223)	
	Q2	1.108	(0.875, 1.403)	
	Q3	1.448	(1.149, 1.824)	
	Q4 (most)	1.469	(1.196, 1.804)	
**Education**				
higher education		1.000	Reference	
high school		1.020	(0.912, 1.142)	
junior high school		1.150	(1.025, 1.290)	
**Occupation**				
white collar		1.000	Reference	
grey collar		1.146	(1.023, 1.284)	
Agriculture		1.203	(1.077, 1.344)	
blue collar		1.209	(1.093, 1.338)	
not working (men)		2.283	(1.945, 2.680)	
not working (women)		1.692	(1.482, 1.931)	
**Cohabitating**				
with others including spouse		1.000	Reference	
with others but not including spouse		1.319	(1.226, 1.418)	
living alone		1.339	(1.144, 1.566)	
**Model Statistics**				
Theta		0.0000		
Explained geographical variance		100.0%		
p value for Theta = 0		1.000		
Log likelihood		−15,260.0		
AIC		30,596.1		
Difference of AIC		494		

Estimate: estimate hazard ratios for the factors and estimated values for the model statistics (Model 5A: adjusted by age, sex, public health centre district, histories of diabetes and hypertension, and body mass index); Qs  =  Quartiles, CI  =  confidence interval; trend p: p value of trend test; Explained geographical variance: percentage of reduction in Theta of fitted models compared to Model 0 shown in Table 2; Difference of AIC: the subtraction of the AIC of the fitted model from the AIC of Model 0 shown in Table 2.

**Table 5:** Hazard ratios and model statistics of the shared frailty survival model with the interaction terms of the dichotomised areal factors (Model 5B).

Factor and category		Estimate	(95% CI)
**Population density × deprivation**			
Population density	Deprivation		
Lower (Q1 and Q2)	Less (Q1 and Q2)	1.000	Reference
	More (Q3 and Q4)	1.035	(0.947, 1.130)
Higher (Q1 and Q2)	Less (Q1 and Q2)	1.009	(0.927, 1.099)
	More (Q3 and Q4)	1.327	(1.162, 1.515)
**Education**			
higher education		1.000	Reference
high school		1.019	(0.910, 1.140)
junior high school		1.141	(1.017, 1.279)
**Occupation**			
white collar		1.000	Reference
grey collar		1.146	(1.023, 1.284)
agriculture		1.181	(1.077, 1.344)
blue collar		1.209	(1.093, 1.338)
not working (men)		2.291	(1.945, 2.680)
not working (women)		1.692	(1.482, 1.931)
**Cohabitating**			
with others including spouse		1.000	Reference
with others but not including spouse		1.315	(1.223, 1.415)
living alone		1.339	(1.145, 1.566)
**Model Statistics**			
Theta		0.0000	
Explained geographical variance		100.0%	
p value for Theta = 0		0.497	
Log likelihood		−15,266.8	
AIC		30,585.6	
Difference of AIC		504.0	

Estimate: estimated hazard ratios for the factors and estimated values for the model statistics (Model 5B: adjusted by age, sex, public health centre district, histories of diabetes and hypertension, and body mass index); Qs  =  Quartiles, CI  =  confidence interval; Explained geographical variance: percentage of reduction in Theta of fitted models compared to Model 0 shown in Table 2; Difference in AIC: the subtraction of AIC of fitted model from AIC of Model 0 shown in Table 2.

Table 3 shows the estimated results of the models that controlled for further confounding and mediating factors (Models 3 and 4). For the two areal factors, HR gradients among the quartile groups were almost unchanged, even after adjusting for initial health condition (Model 3). In this model, the most deprived and most densely inhabited neighbourhoods have about 1.160 and 1.205 times higher HRs for all-cause mortality compared with the least deprived and least densely inhabited ones, respectively. It should be noted that the extra variation in survival rates between neighbourhoods was almost negligible in Models 2 and 3. This indicates that the two areal factors almost explain the extra variation in mortality between neighbourhoods.

The results of Model 4, which included health behaviours as mediating factors, suggest that adjusting for these behaviours reduced the HR variation of individualistic socio-economic status and household structure. For example, there was no significant difference in HR among the three education categories (higher education, high school, and junior high school) in Model 4. In contrast, the HR gradient of the two areal factors was unchanged in terms of point estimates and 95% confidence intervals.

Table 4 shows the estimated HRs from the model with the interaction term of the two areal factors (Model 5A). A trend test of the HRs of the deprivation quartile groups was conducted for each quartile group of population density. Although the model is over-parameterised (AIC of Model 5A is larger than that of Model 3), the results indicate that the HR gradient among the deprivation groups was steeper for groups of larger population density. The trend test of HR gradient was significant at the 5% level only for the group with the largest population density.

The estimated HRs from Model 5A suggest that high HRs are mainly found in neighbourhoods with both high population density (third and fourth quartiles) and high deprivation (third and fourth quartiles). We then integrated the quartile groups of areal deprivation and population density into two categories, Lower/Less (first and second quartiles) and Higher/More (third and fourth quartiles), thereby yielding Model 5B. Model 5B uses the simplified (dichotomised) areal terms, while the other terms remained the same as in Model 5A. The results (Table 5) indicate that the simplified model was superior to Model 3 in terms of AIC, and the interaction of the two areal factors was significant. This reinforced the observation that residents living in a neighbourhood with high deprivation and high population density at baseline had a greater mortality risk. The addition of behavioural variables to Model 5B, as was done in Model 4, did not significantly change the estimated HRs for areal factors.

In these fitted models, most of the interaction terms of each explanatory variable with sex were insignificant and not included in the models, except in the case of age (Models 1–5), hypertension (Models 3–5), and alcohol consumption (Model 4). We observed no significant cross-level interactions between individual and areal contextual variables at the 5% significance level.

## Discussion

The present results indicate that neighbourhood contextual factors contribute to variations in all-cause mortality among the middle-aged population of non-metropolitan settings in Japan. People residing in neighbourhoods with a higher population density and higher ADI showed increased risk of all-cause mortality. Our results reveal that neighbourhood contextual factors contribute to health disparities in Japan, as they do in Western countries [6]–[12]. While Ito et al. [36] already reported an association between socio-economic status and all-cause mortality using the JPHC data, our results indicate that areal contextual factors are additional determinants of mortality for the middle-aged Japanese population. It is worthwhile to note that we controlled for the health conditions of individuals at baseline when analysing the effects of neighbourhood conditions on mortality.

In previous social epidemiology research, areal effects of neighbourhood conditions were commonly interpreted in terms of social and material dimensions. For example, one contributing social factor could be the lack of social norms against unhealthy behaviours, such as heavy drinking and smoking, in deprived areas. Population density (urbanicity) may further impact this tendency by increasing anonymity and decreasing common interest among residents. Indeed, our analysis indicated that areal deprivation was most influential on health in the most densely inhabited urbanised parts.

However, while the major health-related behaviours (smoking, alcohol intake, and sports activity) are associated with socio-economic status at the individual level, these common behavioural factors did not attenuate the observed association between areal factors and mortality in this study. Therefore, we need to consider other possible factors that mediate the relationship between living area and mortality.

Recently, several cross-sectional studies in Japan on social capital employed aggregated variables of social trust (i.e. cohesive attitudes or interactions among neighbours) for each areal unit, demonstrating that rich community-level social capital was positively associated with better health in most cases [21]–[23]. However, these studies were inconsistent in their use of indicators to measure social capital, and more importantly, it was not explained how the studied areas with better/worse community health were geographically situated in terms of deprivation and urbanicity. Hanibuchi et al. [37] later explored the contextual determinants of community-based social capital in a region covering suburban and rural settlements in Aichi prefecture, and found that older rural settlements tended to have higher social capital indicators than did recently developed residential areas. Furthermore, elderly people living in rural areas tend to have a lower incidence of depressive symptoms compared with those living in urban areas in Japan [38]. Thus, the rural advantage of a cohesive and supportive social milieu may be responsible for the tendency in this study for less-populated residential areas to be healthier than densely populated ones. In addition, social environmental effects on health appear rooted in areal deprivation. Tabuchi et al. [24] demonstrated that ‘address discrimination’ towards disadvantaged people living in deprived areas [39] is applicable to Japanese deprived neighbourhoods in Osaka city. They observed that higher perceived place-based discrimination is associated with worse mental health among residents in these deprived areas. Long-term accumulation of area-based mental health problems could explain the areal deprivation effects on mortality, even in non-metropolitan settings.

Regarding the material and physical dimensions of areal contextual effects, Chaix et al. [3] and Meijer et al. [4] suggested that environmental pollution and availability of community resources might play a mediatory role. In another study using JPHC data, an elevated risk of cardiovascular disease in PHC districts with higher levels of particulate matter (PM) in the air [40] was observed, although geographical variations in PM levels within a PHC district might be too small to cause a real geographical variation in mortality. While the housing market may lead to the accumulation of socially disadvantaged people [in terms of housing conditions, access to healthcare, and the availability of other daily use facilities (e.g. parks and shopping places)] in less favourable residential areas, no study has yet measured such inequalities in physical environmental factors in relation to urbanicity and deprivation in Japan. Additionally, high population density may promote physical activity [26]; thus, urbanicity may influence both the beneficial and harmful aspects of neighbourhood conditions. As such, we need further research to clarify the mediating processes of areal contextual factors by investigating how areal deprivation and urbanicity are related to more detailed aspects of neighbourhood condition.

There are several caveats to our analytical results in this study. First, our study is limited in terms of the data availability for constructing areal factors: there is a five-year gap between the census data and the baseline survey. Second, the areal indicators used may reflect individual factors that were not explored in this study. For example, high population density might be related to overcrowded housing conditions, which would be considered an individual-level deprivation [3]. In this study, we could not control for housing conditions at the individual level because such information was not available at baseline. Third, we only analysed data from a middle-aged sample; thus, the applicability of these findings to younger populations has not been determined. Deaths from suicide and injuries are more prevalent in younger relative to middle-aged and elderly people. Fukuda et al. [41] conducted an ecological study using municipality-level mortality data and reported that the relative contribution of these causes of death to regional socio-economic inequalities in mortality in people aged less than 75 years in Japan increased between the 1970s and the 1990s, while that of stroke mortality decreased. Thus, younger people may experience greater socio-economic inequalities in mortality at the areal and/or individual level compared to middle-aged or elderly people. Fourth, we need further clarification about the associations between areal characteristics and mortality based on the interaction between living area and health [39]. Since people tend to live in different geographical settings and occupy varying socio-economic statuses throughout life, their health may be differentially impacted at different time periods. While we just considered areal indicators at one time point near the baseline, White et al. [42] compared associations of elderly mortality with areal and individual socio-economic characteristics at three different time points. An additional study should be conducted to evaluate the effects of migration history and variations in individual socio-economic status on health [43], [44]. Finally, our data only covered four districts, and the sample size is not large enough to extend our findings to the general population of Japan. Thus, we should verify our findings by including more regions, particularly metropolitan areas.

It is, however, notable that the census-based areal factors used in this study can be applied to other regions of Japan to verify the present findings. Areal deprivation and population density may represent useful indicators by which to base the monitoring of health gaps between neighbourhoods. The national policy of public health in Japan intends to promote population health by creating health-supportive environments such as enhancing walkability and healthy food access [45]. Although the new policy [27] intends to reduce the health gaps between social statuses and regions, there is still a lack of evidence regarding how such specific health promotion can reduce the health gaps between neighbourhoods. Further research should integrate knowledge of how neighbourhoods shape specific health aspects and how modifications of neighbourhoods may affect social gradation in health on both the individual and areal levels in the context of Japanese society.

## Supporting Information

Appendix S1 **The Japanese census-based deprivation index.**
(PDF)

Appendix S2 **Occupational classification.**
(PDF)

Appendix S3 **The member list of JPHC Study Group.**
(PDF)
