# Supplementary material for: Associations of All-Cause Mortality with Census-Based Neighbourhood Deprivation and Population Density in Japan: A Multilevel Survival Analysis
Source: PLoS One. 2014 Jun 6;9(6):e97802. doi: 10.1371/journal.pone.0097802 (PMC4048169; doi:10.1371/journal.pone.0097802)
Supplement: Appendix S1 — The Japanese census-based deprivation index. (PDF) [file pone.0097802.s001.pdf]

Appendix S1 The Japanese census-based deprivation index

The areal deprivation index (ADI) was calculated using deprivation-related census variables in each CA, as follows:

$ADI_i = k \times (2.99 \times \textit{proportion of old couple households}_i + 7.57 \times \textit{proportion of old single households}_i + 17.4 \times \textit{proportion of single-mother households}_i + 2.22 \times \textit{proportion of rent houses}_i + 4.03 \times \textit{proportion of sales and service workers}_i + 6.05 \times \textit{proportion of agricultural workers}_i + 5.38 \times \textit{proportion of blue caller workers}_i + 18.3 \times \textit{unemployment rate}_i),$

where *i* is an area index and *k* refers to a positive constant. The value of *k* was originally assigned by making a synthetic estimate of the number of 'poverty' households in an area. However, assigning any positive number produces the same order of CAs in terms of their deprivation score. The relative position can be used to create quartile groups of areal units in terms of areal deprivation. The weights of the ADI on the census-based variables in the above equation were taken from the estimated odds ratios in a logistic regression analysis used to predict 'poverty households', using microdata from Japanese social surveys measuring poverty, as described below.

As in previous studies where this method was used to calculate deprivation [1,2], the Japanese deprivation index [3] used here defined a 'poverty household' as a household of low socio-economic position, both objectively and subjectively; that is, a household with an equalized annual household income below half of the national median and that had a reference occupant who identified with being of low social class (i.e. selected one of the bottom two choices of a 5-point 'top-bottom self-placement' question), respectively. The income was adjusted by regional differences in housing cost and commodity prices according to the public assistance calculation system. Data for the weights of the Japanese ADI were taken from the Japanese General Social Survey (JGSS) cumulative data 2000–2003, which is a micro dataset of nationally representative samples of adult Japanese. The JGSS estimate of the national poverty rate based on the abovementioned definition was 8.62% for the 2000–2003 period. We cannot find any other similar microdata sources collected before 2000; data from the 1990s would have been ideal. The JGSS data were submitted to a logistic regression analysis, the results of which we used to derive the ADI weights in order to predict poverty households. The large weights in the above ADI equation indicate that the census variables were most likely associated with

poverty. Only significant coefficients from the logistic regression at the 5% level were used for the weighting. The weights used here were slightly different from those used in the original paper [3] because of some modifications that we made to missing cases and occupational categories in the JGSS data, which were adapted to fit the categories of the JPHC Study. However, there were no significant differences in these weights, meaning that they likely had little impact on our results.

The abovementioned subjective and objective socio-economic status measures have been shown to be sensitive to self-rated health within the Japanese population [4]. Furthermore, the ADI was tested against ecological datasets of all-cause and various cancer mortalities at the municipal level across Japan and showed consistent positive relationships to most of the mortality indices [3]. Thus, the ADI is likely a suitable reflection of deprivation for our sample.

1. Dorling D, Rigby J, Wheeler B, Ballas D, Thomas B, et al. (2007) Poverty and wealth across Britain 1968 to 2005. Bristol: The Policy Press, 111p.
2. Pornet C, Delpierre C, Dejardin O, Grosclaude P, Launay L, et al. (2012) Construction of an adaptable European transnational ecological deprivation index: The French version. J Epidemiol Commun Health 66: 982–989.
3. Nakaya T (2011) Evaluating socioeconomic inequalities in cancer mortality by using areal statistics in Japan: A note on the relation between the municipal cancer mortality and the areal deprivation index. P I Stat Math 59: 239–265.
4. Hanibuchi T, Nakaya T, Murata C (2012) Socio-economic status and self-rated health in East Asia: a comparison of China, Japan, South Korea and Taiwan. Eur J Pub Health 22: 47–52.
