# Supplementary material for: Associations of All-Cause Mortality with Census-Based Neighbourhood Deprivation and Population Density in Japan: A Multilevel Survival Analysis
Source: PLoS One. 2014 Jun 6;9(6):e97802. doi: 10.1371/journal.pone.0097802 (PMC4048169; doi:10.1371/journal.pone.0097802)
Supplement: Appendix S2 — Occupational classification. (PDF) [file pone.0097802.s002.pdf]

Appendix S2: Occupational classification

We recoded JSOC-based major occupational categories into four classes that are also used for the areal deprivation index. These are roughly corresponding to common categories between JSOC and ISCO, which are shown as the following table.

Table S1 Category correspondences among JSOC, ISCO and classification used in this study

JSOC (3rd and 4th rev)	ISCO-88	Classification used in this study
<Administrators> Administrative and managerial workers	Legislators, senior officials and managers	White collar
<Professionals> Specialist and technical workers	Professionals<br>Technicians and associate professionals	
<Office clerks> Clerical workers	Clerks	
<Sales clerks> Sales workers<br><Service workers> Service workers<br><Security workers> Security workers	Service workers and shop and market sales workers	Grey collar
<Agricultural workers> Agriculture forestry and fishery workers	Skilled agricultural and fishery workers	Agricultural
<Transport and communication workers> Transport and communication workers<br><Manual labourers> Production process and related workers	Craft and related trades workers<br>Plant and machine operators and assemblers<br>Elementary occupations	Blue collar
Workers not classifiable by occupation		NA
	Armed forces	NA

Notes: JSOC: Japan Standard Occupation Classification, ISCO: International Standard Classification of Occupation.

The occupational titles shown in parentheses <> in the column of JSOC are the shortened names used in this manuscript.

The relation of categories between JSOC and ISCO was obtained from Statistical Bureau information (http://www.stat.go.jp/index/seido/shokgyou/hik_h21.htm).
