# Supplementary material for: Associations of All-Cause Mortality with Census-Based Neighbourhood Deprivation and Population Density in Japan: A Multilevel Survival Analysis
Source: PLoS One. 2014 Jun 6;9(6):e97802. doi: 10.1371/journal.pone.0097802 (PMC4048169; doi:10.1371/journal.pone.0097802)
Supplement: Appendix S3 — The member list of JPHC Study Group. (PDF) [file pone.0097802.s003.pdf]

Appendix S3: The member list of JPHC Study Group

Members of the Japan Public Health Center-based Prospective Study (JPHC Study, principal investigator: S. Tsugane) Group are: S. Tsugane, N. Sawada, S. Sasazuki, M. Iwasaki, T. Shimazu, T. Yamaji, and T. Hanaoka, National Cancer Center, Tokyo; J. Ogata, S. Baba, T. Mannami, A. Okayama, and Y. Kokubo, National Cerebral and Cardiovascular Center, Osaka; K. Miyakawa, F. Saito, A. Koizumi, Y. Sano, I. Hashimoto, T. Ikuta, Y. Tanaba, H. Sato, Y. Roppongi, and T. Takashima, Iwate Prefectural Ninohe Public Health Center, Iwate; Y. Miyajima, N. Suzuki, S. Nagasawa, Y. Furusugi, N. Nagai, Y. Ito, S. Komatsu and T. Minamizawa, Akita Prefectural Yokote Public Health Center, Akita; H. Sanada, Y. Hatayama, F. Kobayashi, H. Uchino, Y. Shirai, T. Kondo, R. Sasaki, Y. Watanabe, Y. Miyagawa, Y. Kobayashi, M. Machida, K. Kobayashi and M. Tsukada, Nagano Prefectural Saku Public Health Center, Nagano; Y. Kishimoto, E. Takara, T. Fukuyama, M. Kinjo, M. Irei, and H. Sakiyama, Okinawa Prefectural Chubu Public Health Center, Okinawa; K. Imoto, H. Yazawa, T. Seo, A. Seiko, F. Ito, F. Shoji and R. Saito, Katsushika Public Health Center, Tokyo; A. Murata, K. Minato, K. Motegi, T. Fujieda and S. Yamato, Ibaraki Prefectural Mito Public Health Center, Ibaraki; K. Matsui, T. Abe, M. Katagiri, M. Suzuki, K. and Matsui, Niigata Prefectural Kashiwazaki and Nagaoka Public Health Center, Niigata; M. Doi, A. Terao, Y. Ishikawa, and T. Tagami, Kochi Prefectural Chuo-higashi Public Health Center, Kochi; H. Sueta, H. Doi, M. Urata, N. Okamoto, and F. Ide and H. Goto, Nagasaki Prefectural Kamigoto Public Health Center, Nagasaki; H. Sakiyama, N. Onga, H. Takaesu, M. Uehara, T. Nakasone and M. Yamakawa, Okinawa Prefectural Miyako Public Health Center, Okinawa; F. Horii, I. Asano, H. Yamaguchi, K. Aoki, S. Maruyama, M. Ichii, and M. Takano, Osaka Prefectural Suita Public Health Center, Osaka; Y. Tsubono, Tohoku University, Miyagi; K. Suzuki, Research Institute for Brain and Blood Vessels Akita, Akita; Y. Honda, K. Yamagishi, S. Sakurai and N. Tsuchiya, University of Tsukuba, Ibaraki; M. Kabuto, National Institute for Environmental Studies, Ibaraki; M. Yamaguchi, Y. Matsumura, S. Sasaki, and S. Watanabe, National Institute of Health and Nutrition, Tokyo; M. Akabane, Tokyo University of Agriculture, Tokyo; T. Kadowaki and M. Inoue, The University of Tokyo, Tokyo; M. Noda and T. Mizoue, National Center for Global Health and Medicine, Tokyo; Y. Kawaguchi, Tokyo Medical and Dental University, Tokyo; Y. Takashima and Y. Yoshida, Kyorin University, Tokyo; K. Nakamura and R. Takachi, Niigata University, Niigata; J. Ishihara, Sagami Women's University, Kanagawa; S. Matsushima and S. Natsukawa, Saku General Hospital, Nagano; H. Shimizu, Sakihae Institute, Gifu; H. Sugimura, Hamamatsu University School of Medicine, Shizuoka; S. Tominaga, Aichi Cancer Center, Aichi; N. Hamajima, Nagoya University, Aichi; H. Iso and T. Sobue, Osaka University, Osaka; M. Iida, W. Ajiki, and A. Ioka, Osaka Medical Center for Cancer and Cardiovascular Disease, Osaka; S. Sato, Chiba Prefectural Institute of Public Health, Chiba; E. Maruyama, Kobe University, Hyogo; M. Konishi, K. Okada, and I. Saito, Ehime University, Ehime; N. Yasuda, Kochi University, Kochi; S. Kono, Kyushu University, Fukuoka; S. Akiba, Kagoshima University, Kagoshima.
